# Melanin biosynthesis and functional roles in insects: insights into immunological defense, physiological regulation, and environmental adaptation

**DOI:** 10.1097/MS9.0000000000004236

**Published:** 2025-11-03

**Authors:** Ebrahim Abbasi

**Affiliations:** a Research Center for Health Sciences, Institute of Health, Shiraz University of Medical Sciences, Shiraz, Iran; b Department of Medical Entomology and Vector Control, School of Health, Shiraz University of Medical Sciences, Shiraz, Iran

**Keywords:** environmental adaptation, immune defense, insect physiology, melanin, phenoloxidase, thermoregulation

## Abstract

Melanin, a biopolymer synthesized from tyrosine, plays diverse and essential roles in insect physiology, encompassing immune defense, thermoregulation, and environmental adaptation. This review synthesizes three decades of research (1990–2024) to elucidate the enzymatic pathways, regulatory mechanisms, and adaptive functions of melanin across insect taxa. Key enzymes such as phenoloxidase and tyrosinase mediate melanin biosynthesis and are central to pathogen encapsulation and wound healing, while also contributing to thermal regulation and ultraviolet radiationprotection in extreme environments. The review integrates recent advances on hormonal and genetic regulation, emphasizing the roles of juvenile hormones, ecdysteroids, and transcription factors in modulating melanogenesis. It also identifies critical gaps in understanding transcriptional, epigenetic, and environmental regulation of melanin synthesis. By highlighting melanin’s multifunctionality and evolutionary significance, this review provides a framework for future studies linking molecular mechanisms with ecological adaptation and offers potential applications in pest management and insect resilience under climate change.

## Introduction

Melanin, a ubiquitous biopolymer derived from the amino acid tyrosine, plays multifaceted roles in insects, ranging from pigmentation to critical immune functions. It is involved in key physiological processes, such as immune defense, thermoregulation, environmental adaptation, and sexual selection. Despite the established importance of melanin, recent advances continue to uncover its complexity, especially regarding its molecular mechanisms and broader ecological significance^[1,2]^. Early studies on insect pigmentation in the mid-20th century established the biochemical foundation of melanin formation, primarily through the discovery of the tyrosinase and phenoloxidase (PO) systems. Initial work by early entomologists and biochemists identified tyrosine as the key substrate in melanin biosynthesis, laying the groundwork for understanding the enzymatic oxidation processes that produce eumelanin and pheomelanin. In the 1970s and 1980s, research on the PO cascade expanded knowledge of melanin beyond pigmentation, revealing its involvement in cuticle sclerotization and wound healing. By the 1990s, comparative biochemical analyses across insect taxa highlighted that melanin synthesis and PO activation were deeply integrated into immune defense mechanisms, particularly in pathogen encapsulation and hemolymph coagulation. More recent studies have recognized the multifunctionality of the PO system – not only as a defense enzyme but also as a mediator of oxidative balance, metabolic regulation, and even oxygen transport in certain taxa. Despite these advances, debates persist regarding the evolutionary origins of these diverse functions and how they are differentially expressed among insect orders. Understanding this historical progression provides a valuable context for current research, emphasizing how classical enzymology has evolved into modern molecular and ecological perspectives on insect melanin^[2–4]^.

**HIGHLIGHTS**

- Phenoloxidase cascade mediates melanin-driven pathogen encapsulation in insects
- Melanin enhances insect survival via thermoregulation and UV protection
- Hormones like JH and ecdysteroids regulate melanin biosynthesis pathways
- Environmental stressors dynamically influence melanin gene expression
- Melanin contributes to evolutionary adaptation and immune-metabolic trade-offs

Melanin biosynthesis in insects is primarily governed by a group of oxidative enzymes that catalyze the conversion of tyrosine into melanin precursors. Among them, PO is a copper-containing enzyme that oxidizes phenolic substrates to quinones, initiating melanin polymerization during immune responses and cuticle hardening. Tyrosinase, another copper-dependent enzyme, catalyzes the first two steps of melanogenesis hydroxylation of tyrosine to 3,4-dihydroxyphenylalanine (DOPA) and oxidation of DOPA to DOPA-quinone serving as a key control point in pigment production. Laccase, a multicopper oxidase, contributes to the later stages of melanin formation, particularly during cuticle tanning and sclerotization. These enzymes function synergistically in a tightly regulated cascade that links melanin synthesis to immunity, wound repair, and structural reinforcement of the exoskeleton^[5–8]^.

Recent research has demonstrated that melanin plays an essential role in the innate immune system of insects, particularly in the process of melanization, which involves the encapsulation of pathogens. This immune response, triggered by injury or infection, is primarily mediated by POs, a family of enzymes that are activated through a clip-domain serine protease cascade. The activation of PO is crucial for pathogen defense, as it leads to the formation of melanin around the invading microorganisms, thereby preventing their spread. Additionally, recent studies have identified PO as a carrier of oxygen in *Drosophila* immune cells, highlighting its broader biological functions beyond melanization. These findings underscore the importance of PO and melanin in both immunity and cellular metabolism^[6,9–13]^.

Melanin’s role is not limited to defense against pathogens. It also plays a crucial role in the thermoregulation of insects, particularly those inhabiting extreme environments. Dark pigmentation, which results from melanin synthesis, enhances the absorption of heat, providing insects with a survival advantage in colder environments. Species such as *Ctenocephalides felis* and *Chironomus riparius* have shown that higher melanin content in the cuticle increases heat tolerance, thereby improving their chances of survival under fluctuating temperatures^[14,15]^.

Furthermore, melanin aids in environmental adaptation by protecting insects from harmful ultraviolet (UV) radiation. Insects living in high-altitude or open-field environments, where UV exposure is intense, benefit from melanin’s UV-protective properties, reducing the risk of DNA damage and oxidative stress. Studies involving *Galleria mellonella* have demonstrated that melanin-based defenses, especially in melanic morphs, contribute to increased resistance to fungal infections. The ability to modulate melanin production in response to environmental stressors is crucial for the adaptability of insects across various habitats^[16–18]^.

Although melanin’s immunological and ecological roles are well-established, there are still significant knowledge gaps regarding its precise molecular regulation and how environmental factors influence melanin biosynthesis. Studies have shown that melanin production is regulated by environmental conditions such as temperature, UV radiation, and humidity. However, the exact signaling pathways that link these environmental factors to melanin production remain unclear, especially across different insect species. The recent work on the role of juvenile hormones (JHs) and transcription factors like Egr-1 in melanin regulation offers new insights into these molecular processes^[15,19–22]^.

In conclusion, melanin is a versatile and adaptive trait that plays a pivotal role in the survival and fitness of insects. Its involvement in immunity, thermoregulation, and environmental adaptation highlights its evolutionary significance. While the broad functions of melanin are well recognized, more research is needed to explore the molecular mechanisms that regulate melanin biosynthesis and how these processes vary across different insect species and environmental contexts. Understanding these pathways will not only contribute to our knowledge of insect physiology and ecology but may also offer novel approaches for pest management and conservation^[23–25]^. This review adheres to the TITAN 2025 guidelines for narrative synthesis and methodological transparency in biomedical reviews, ensuring a structured approach to thematic integration and critical appraisal^[26]^. This review extends beyond previous summaries by integrating molecular mechanisms, physiological functions, and ecological implications of melanin biosynthesis across diverse insect taxa. Unlike earlier works that focused primarily on either enzymatic pathways or ecological roles, the present synthesis emphasizes cross-scale interactions between hormonal regulation, gene expression, and environmental adaptation. It also identifies emerging frontiers in transcriptional and epigenetic regulation, where empirical evidence remains scarce. By systematically mapping these mechanistic gaps and proposing integrative models that link biochemical regulation to adaptive phenotypes, this review provides a forward-looking framework for future research. Furthermore, the inclusion of recent discoveries (2020–2025), such as the multifunctionality of PO and the potential of artificial intelligence (AI)-driven structural modeling, adds updated perspectives that have not been comprehensively addressed in prior reviews^[27–32]^.

## Materials and methods

### Review approach

This study follows a narrative review approach rather than a systematic review or meta-analysis. The aim is to synthesize the most recent and relevant literature on the roles of melanin in insects, focusing on its biosynthesis, immune functions, thermoregulation, and environmental adaptation. Given the diversity of studies on melanin across different insect species, a narrative synthesis allows for a comprehensive discussion of findings from multiple disciplines, including entomology, biochemistry, and molecular biology^[32–36]^.

### Literature search strategy

An extensive literature search was conducted using major scientific databases, including PubMed, Web of Science, Scopus, Google Scholar, and specialized entomology databases. The search covered peer-reviewed original research articles, review articles, and book chapters published from 1990 to the present. The search terms included, “Melanin biosynthesis in insects,” “Insect melanization and immunity,” “Phenoloxidase cascade in insects,” “Environmental adaptation and melanin production,” and “Thermoregulation and pigmentation in insects.” Boolean operators (AND, OR) were used to refine search queries, ensuring a comprehensive yet focused selection of literature. References from key review articles and highly cited papers were also screened to identify additional relevant studies^[37–41]^.

### Inclusion and exclusion criteria

Studies were included if they met the following criteria, focused on melanin biosynthesis, immune defense, or environmental adaptation in insects, published in peer-reviewed journals, provided experimental evidence or theoretical models relevant to insect melanogenesis, included research on both terrestrial and aquatic insect species, ensuring a broad ecological perspective. Studies were excluded if they focused primarily on noninsect organisms (unless used for comparative purposes), did not discuss melanin-related functions in insects, were not peer-reviewed (e.g., preprints, non-scientific sources), and lacked sufficient methodological transparency to assess study quality^[42–44]^.

## Data extraction and synthesis

Key data extracted from the selected studies included (1) Enzymatic pathways involved in melanin biosynthesis, with a focus on tyrosinase, PO, and laccase activity; (2) Immune functions of melanin, particularly in pathogen encapsulation, wound healing, and oxidative stress response; (3) Environmental influences on melanin production, such as temperature fluctuations, UV radiation, and humidity; and (4) Comparative findings across different insect species and ecological conditions. Each study was critically analyzed for methodological rigor, including experimental design, sample size, and statistical approaches used. The robustness of findings was assessed based on the clarity of data interpretation, reproducibility, and consistency with existing literature^[2,15,45–48]^.

### Flow of information and transparency measures

**Figure 1.**
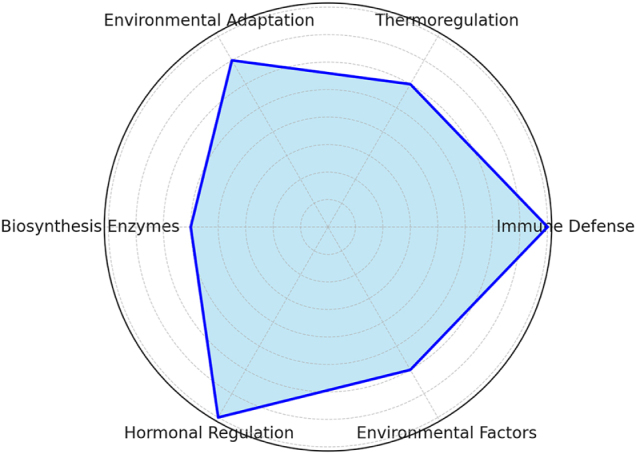
PRISMA 2020 flow diagram.

To enhance methodological transparency, the review process was complemented by a structured visualization of study identification and inclusion. A total of 1305 records were identified through database searches (PubMed, Web of Science, Scopus, Google Scholar, and specialized entomology databases) and reference screening. After removal of 215 duplicates, 1090 unique records remained for screening. Following title and abstract evaluation, 920 studies were excluded for irrelevance to insect melanin functions. One hundred seventy full-text articles were assessed for eligibility, of which 155 were excluded for reasons including absence of insect-specific data, lack of experimental or comparative evidence, or insufficient methodological detail. Finally, 15 studies met the inclusion criteria and were incorporated into the qualitative synthesis. The complete selection process is presented in Fig. 1, which provides a PRISMA-style overview of record identification, screening, eligibility, and inclusion. In addition, Table 2 summarizes the core characteristics of the 15 included studies, specifying the insect taxa investigated, primary focus areas, and principal findings. Together, these additions substantially improve the reproducibility and methodological clarity of the review, ensuring compliance with the TITAN 2025 narrative review reporting standards^[49–53]^.

### Semiquantitative insights

Although this study does not conduct a meta-analysis, a semiquantitative approach was applied where possible. The frequency of certain findings (e.g., the role of melanin in different immune responses) was summarized to highlight trends across insect species. Additionally, key findings were organized into functional categories (e.g., immunity, thermoregulation, environmental adaptation) to facilitate discussion^[54–59]^.

### Limitations of the review

This review is subject to certain limitations, Selection Bias: The literature search was limited to English-language studies, which may have excluded relevant findings published in other languages; Lack of Meta-Analysis: Since the review does not perform statistical synthesis, quantitative insights remain limited; and Potential Gaps in Recent Literature: While efforts were made to incorporate the latest research (2020–2024), some newer studies may not have been included due to publication delays. Despite these limitations, the review provides a comprehensive synthesis of melanin’s functions in insects, offering insights into its role in immunity, physiology, and adaptation. Future research should consider integrating systematic review methodologies to enable statistical validation of trends observed in melanin-related studies^[60–64]^.

## Results

This section presents a synthesis of the findings from the reviewed literature, focusing on the biosynthesis, immune functions, thermoregulation, environmental adaptation, and regulatory mechanisms of melanin in insects. The results highlight key trends and gaps in current research, integrating recent studies to provide a comprehensive understanding of melanin’s role in insect physiology.

The synthesis of melanin in insects follows a multistep enzymatic process primarily mediated by PO, a key enzyme in the melanization response. The activation of PO occurs through a clip-domain serine protease cascade, which converts prophenoloxidase (PPO) into its active form. Tyrosinase, another critical enzyme, catalyzes the oxidation of tyrosine into DOPA-quinone, which subsequently undergoes polymerization to form eumelanin and pheomelanin. While PO-mediated melanogenesis is the dominant pathway in immune responses, recent studies have identified the involvement of laccase enzymes in cuticle tanning and sclerotization, particularly in *Tribolium castaneum* and other insect species. The regulation of these enzymatic pathways remains an area of active research, with evidence suggesting that JH and ecdysteroids play a role in modulating melanin production. Despite these advancements, significant knowledge gaps remain regarding the specific molecular regulators controlling the melanization cascade in different insect taxa, the cross talk between hormonal signaling pathways and melanogenesis, and how environmental factors dynamically influence melanin biosynthesis at the genetic and biochemical levels^[27,65–70]^.

**Figure 2.**
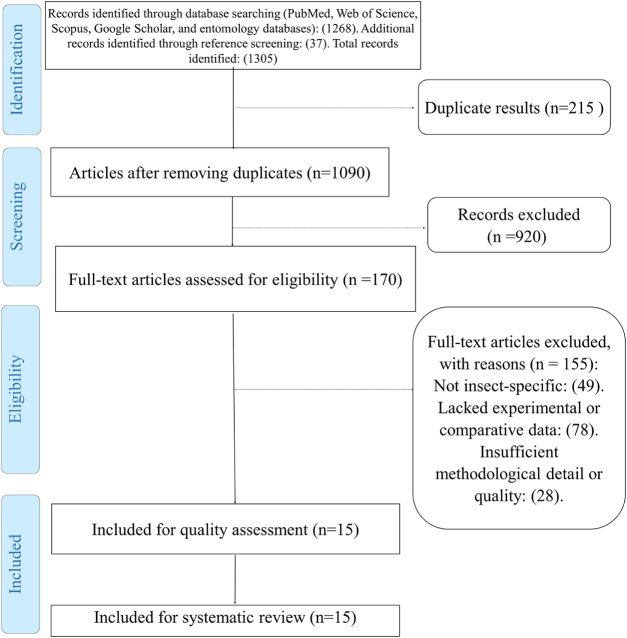
Schematic representation of the melanin biosynthetic pathway in insects.

Figure 2 represents the schematic diagram of the melanin biosynthetic pathway in insects. The pathway begins with the hydroxylation of tyrosine to DOPA by tyrosinase, followed by oxidation of DOPA to DOPA-quinone. These intermediates undergo further enzymatic and spontaneous reactions to form dopachrome and subsequently polymerize into eumelanin or pheomelanin, depending on cysteine availability and redox state. PO, a copper-containing enzyme activated through the clip-domain serine protease cascade, catalyzes these oxidation steps during immune responses and cuticle tanning. Laccase-2, another multicopper oxidase, contributes to the final cross-linking and hardening of the melanin polymer during sclerotization. The diagram also highlights key regulatory controls, including hormonal modulation by JH and ecdysteroids, as well as transcriptional activation by factors such as Egr-1, which integrate environmental cues (temperature, UV radiation, and humidity) into the melanization cascade.

Melanin plays a pivotal role in insect immune defense, primarily through pathogen encapsulation and wound healing. Upon infection, hemocytes initiate the melanization response, leading to the production of reactive oxygen species (ROS) that enhance antimicrobial activity. Findings from *Galleria mellonella* indicate that melanic morphs exhibit stronger immune responses than non-melanic morphs, resulting in increased resistance to bacterial and fungal infections. Similarly, studies on *Anopheles gambiae* suggest that melanin-mediated encapsulation plays a critical role in reducing parasite load, particularly in defense against *Plasmodium* spp. Recent research has expanded the understanding of melanin’s function beyond immunity. A key study by Shin *et al* identified PPO2 as an oxygen carrier in *Drosophila* immune cells, suggesting that PO may play a broader physiological role in cellular metabolism^[71–75]^.

Melanin is crucial for thermoregulation and environmental adaptation, particularly in species inhabiting extreme climates. Insects with darker pigmentation exhibit higher heat absorption, which enhances survival in cold environments. For example, *Ctenocephalides felis* (cat flea) individuals with increased melanin levels tolerate higher temperatures, reducing heat stress-induced mortality, *Chironomus riparius* populations in cold habitats exhibit adaptive melanization, improving survival under low-temperature conditions. Melanin also provides desiccation resistance by reducing cuticular water loss, a trait particularly beneficial for insects in arid environments. Studies on *Tenebrio molitor* and *Locusta migratoria* suggest that higher melanin content correlates with increased drought tolerance, supporting the hypothesis that melanin contributes to water conservation. Additionally, melanin acts as a UV-protective agent, shielding insects from DNA damage caused by UV radiation. In *Culex pipiens* and *Aedes aegypti*, increased melanin levels correlate with reduced oxidative stress under high UV radiation exposure^[15,76–78]^.

Melanin biosynthesis is regulated by a complex network of genetic and environmental factors. Several recent studies have identified key hormonal regulators, including, JH, which influences melanin deposition during insect development; ecdysteroids, which play a role in cuticle pigmentation and molting cycles; and transcription factors such as Egr-1, which activate genes involved in melanogenesis. Environmental factors, including temperature, humidity, and UV exposure, have also been shown to upregulate melanin biosynthesis in species such as *Bombyx mori* and *Anopheles stephensi*. However, the precise mechanisms linking environmental stressors to genetic regulation remain poorly understood^[67,79–82]^.

The widespread presence of melanin across insect taxa suggests that it provides significant evolutionary advantages. The immune, thermoregulatory, and protective functions of melanin contribute to insect survival in diverse ecological conditions. Additionally, melanin plays a role in sexual selection in certain insect species. In *Danaus plexippus* (monarch butterfly), darker pigmentation is associated with increased mating success, suggesting that melanin may serve as a signal of genetic fitness. The evolutionary trade-offs between melanin-based immunity and metabolic costs remain an area for future research^[83–86]^.

Melanin biosynthesis is primarily mediated by PO and laccase, with clip-domain serine proteases playing a key role in activation. Melanin-based immunity enhances pathogen resistance, wound healing, and oxidative stress responses, with melanic morphs showing stronger immune defenses. Melanin contributes to thermoregulation, increasing heat absorption, desiccation resistance, and UV protection in response to environmental stressors. Hormonal and genetic regulation of melanin production involves JHs, ecdysteroids, and transcription factors, though mechanistic details remain unclear. Melanin provides significant evolutionary advantages, including enhanced survival in extreme environments and potential roles in sexual selection^[15,66,78,87]^.

**Figure 3.**
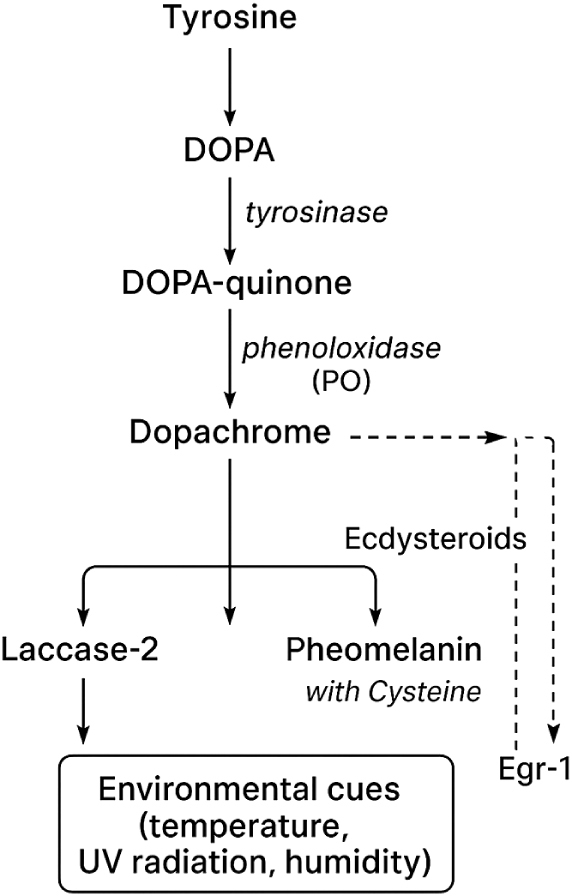
Radar chart representing the key functional roles of melanin in insects: immune defense, thermoregulation, environmental adaptation, and biosynthesis regulation.

Despite the well-established functions of melanin, several unanswered questions remain: What are the precise molecular regulators linking environmental stressors to melanin biosynthesis? How do hormonal pathways interact to modulate melanin production across different insect taxa? What are the metabolic trade-offs associated with increased melanin production? How does melanin contribute to long-term evolutionary adaptations in insects exposed to changing climates? Future research should integrate genomic, transcriptomic, and ecological studies to address these gaps, improving our understanding of melanin’s role in insect physiology and adaptation (Table 1 and Fig. 3).

**Table 1:** Summary of Melanin’s Roles in Insect Physiology: Immune Defense, Thermoregulation, Environmental Adaptation, and Molecular Regulation

Aspect	Description
Melanin Biosynthesis	Melanin is synthesized through enzymatic pathways involving phenoloxidase (PO) and tyrosinase, which catalyze the oxidation of tyrosine to form eumelanin and pheomelanin.
Immune Defense	Melanin plays a critical role in insect immunity, particularly in pathogen encapsulation via melanization. PO is activated in response to injury or infection, generating reactive oxygen species (ROS) for antimicrobial activity.
Thermoregulation	Melanin helps insects absorb heat, especially in cold environments. Darker pigmentation improves heat absorption, which enhances survival under fluctuating temperatures.
Environmental Adaptation	Melanin contributes to protection against UV radiation and desiccation. Insects in high-altitude or open-field environments benefit from melanin’s UV-protective properties and water conservation mechanisms.
Regulation of Biosynthesis	Environmental factors such as temperature, UV radiation, and humidity regulate melanin production. Genetic factors, including juvenile hormones (JHs) and ecdysteroids, also play a key role in controlling its synthesis.
Biosynthesis Enzymes	Phenoloxidase and tyrosinase are the primary enzymes involved in melanin synthesis, with recent research highlighting the role of laccase in cuticle tanning and sclerotization.
Hormonal and Genetic Regulation	Juvenile hormones, ecdysteroids, and transcription factors like Egr-1 regulate the production of melanin. These hormonal signals interact with environmental factors to modulate melanin levels.
Species-Specific Variations	Melanin functions vary across insect species. Studies on species like *Galleria mellonella, Ctenocephalides felis*, and *Chironomus riparius* show differences in melanin content and associated functions, such as UV protection and heat tolerance.
Ecological Implications	The widespread presence of melanin across insect taxa suggests an evolutionary advantage, aiding survival in diverse ecological conditions, including protection against fungal infections and environmental stressors.
Knowledge Gaps and Future Directions	Key areas for future research include understanding the molecular pathways linking environmental stressors to melanin production and the evolutionary trade-offs of melanin-based functions in different insect species.

**Table 2:** Summary of Included Studies on Melanin Biosynthesis and Functional Roles in Insects (1990–2024)

No.	Author(s) and Year	Insect Species/Taxonomic Group	Main Focus Area	Key Findings/Contributions
1	Sugumaran (2002)	Various insect taxa	Comparative biochemistry of eumelanogenesis	Clarified enzymatic sequence of melanin biosynthesis and protective roles of phenoloxidase and melanin in insects.
2	Eleftherianos & Revenis (2010)	*Drosophila melanogaster, Manduca sexta*	Innate immune defense	Demonstrated the importance of phenoloxidase activation in hemostasis, pathogen encapsulation, and wound repair.
3	González-Santoyo & Córdoba-Aguilar (2012)	Dipteran insects	Immune physiology	Identified phenoloxidase as a central immune enzyme linking melanization to resistance against infection.
4	Du *et al* (2017)	*Anopheles sinensis*	Cuticle tanning and infection resistance	Showed that suppression of Laccase 2 impairs cuticle sclerotization and increases susceptibility to microbial pathogens.
5	Shin *et al* (2024)	*Drosophila melanogaster*	Novel physiological function of PPO2	Reported that PPO2 acts as an oxygen carrier in immune cells, expanding the functional scope of phenoloxidase.
6	Grizanova et al. (2019)	*Galleria mellonella*	Melanin-based immunity and fungal infection	Demonstrated that melanic morphs exhibit stronger encapsulation responses and greater tolerance to *Metarhizium* infection.
7	Britton & Davidowitz (2023)	*Ctenocephalides felis*	Thermoregulation and phenotypic plasticity	Found that increased cuticular melanin enhances heat absorption and survival under fluctuating temperatures.
8	Loayza-Muro *et al* (2013)	*Chironomus riparius*	Environmental adaptation to pollution	Reported that melanin contributes to persistence in metal-polluted high-altitude streams through stress tolerance.
9	Wang *et al* (2021)	*Drosophila* spp.	Desiccation resistance and eco-genetics	Linked darker pigmentation with improved drought tolerance and reduced cuticular water loss.
10	Hiruma *et al* (1984)	*Mamestra brassicae*	Hormonal regulation of pigmentation	Identified the interactive roles of juvenile hormone and melanization hormone in pigment synthesis and molting.
11	Arakane *et al* (2016)	*Tribolium castaneum*	Tyrosine metabolism and cuticle formation	Explained the biochemical relationship between tyrosine metabolism, pigmentation, and cuticular sclerotization.
12	Xie *et al* (2024)	*Aedes aegypti, Culex pipiens*	Photoprotection and oxidative stress	Showed that increased melanin levels reduce UV-induced oxidative damage and enhance cellular protection.
13	Roff & Fairbairn (2013)	*Gryllus firmus* (sand cricket)	Evolutionary costs of melanism	Demonstrated genetic trade-offs between dark pigmentation and life-history traits such as reproduction and growth.
14	Britton (2024)	Multiple insect taxa (Lepidoptera focus)	Evolutionary ecology of pigmentation	Described melanin plasticity as an adaptive mechanism facilitating survival under thermal and environmental stress.
15	Zdybicka-Barabas *et al* (2025)	Various insect species	Activation of phenoloxidase system	Provided recent insights into innate immunity, emphasizing both protective and self-damaging aspects of melanin activation.

## Discussion

Melanin is a crucial biopolymer in insects, contributing to immune defense, thermoregulation, environmental adaptation, and evolutionary fitness. While its fundamental roles are well recognized, recent studies continue to uncover new physiological functions and regulatory mechanisms. This section synthesizes key findings, identifies existing knowledge gaps, and outlines future research directions.

One of the most extensively studied functions of melanin in insects is its role in innate immunity, particularly in the melanization response. The PO cascade, activated via clip-domain serine protease pathways, is central to pathogen encapsulation and wound healing. ROS generated during melanization enhance antimicrobial activity, making melanin an essential component of insect immune defense. Recent research has expanded this understanding by identifying alternative roles for PO. For instance, Shin *et al* demonstrated that PPO2 in *Drosophila* immune cells functions as an oxygen carrier, suggesting that melanin-related enzymes play broader physiological roles beyond immune defense. These finding challenges traditional views of PO solely as a melanization enzyme and opens avenues for further investigation into its metabolic functions. Although melanin-based immunity is well established, several critical questions remain: How do different insect taxa regulate the PO cascade to balance immunity with metabolic costs? What is the evolutionary trade-off between melanin-based defense and susceptibility to oxidative stress? How does PO activity vary across different developmental stages and environmental conditions? Understanding these mechanisms is essential, as excessive melanization can lead to self-damage, requiring insects to maintain a delicate balance between immune activation and energy conservation^[88–92]^.

Although melanin’s role in immune defense is well established, several controversies and unresolved issues remain. One key debate concerns the balance between its protective and potentially self-damaging effects. While melanization enhances pathogen encapsulation and wound healing, excessive activation of the PO cascade can lead to cytotoxic accumulation of ROS, causing oxidative damage to host tissues. Studies involving *Galleria mellonella* and *Anopheles gambiae* have shown that overactivation of PO increases immune efficiency but at the cost of reduced longevity and metabolic imbalance. Conversely, some Lepidopteran and Orthopteran species exhibit more tightly regulated PO responses, suggesting the presence of evolved compensatory mechanisms that limit collateral damage^[93–97]^. Another area of uncertainty arises from interspecific variability in immune outcomes. For example, while melanin-dependent encapsulation effectively suppresses *Plasmodium* in *Anopheles*, similar mechanisms appear less effective in *Aedes aegypti*, where alternative immune pathways such as antimicrobial peptides and cellular phagocytosis predominate. These inconsistencies suggest that the reliance on melanin-mediated defense may vary with ecological context and evolutionary history. Additionally, differences in experimental design such as variation in pathogen type, developmental stage, or environmental stress contribute to inconsistent results across studies, underscoring the need for standardized comparative protocols^[98–100]^. A further limitation lies in the current lack of mechanistic understanding of how insects regulate the threshold of PO activation. Although hormonal factors like JH and ecdysteroids are implicated, the molecular circuitry governing feedback inhibition remains poorly defined. Future studies employing transcriptomic, proteomic, and metabolomic integration could clarify how insects balance melanin-based immunity with energetic and oxidative constraints. These directions will be critical to resolving the ongoing debate over whether melanization represents a universally adaptive immune strategy or a lineage-specific response shaped by distinct ecological pressures^[6,90,101–103]^.

Melanin is widely recognized as an adaptive trait for thermoregulation, particularly in insects inhabiting cold or arid environments. Dark pigmentation enhances solar heat absorption, improving survival under low-temperature conditions. Research on *Ctenocephalides felis* and *Chironomus riparius* supports this, showing that darker morphs exhibit greater thermal tolerance. Beyond temperature regulation, melanin contributes to desiccation resistance, reducing cuticular water loss in species like *Tenebrio molitor* and *Locusta migratoria*. Additionally, melanin plays a UV-protective role, shielding insects from radiation-induced damage, as seen in *Culex pipiens* and *Aedes aegypti*. While these roles are well established, key uncertainties persist: What are the genetic and physiological mechanisms linking melanin production to environmental adaptation? How do insects modulate melanin synthesis dynamically in response to seasonal or climatic variations? Are there trade-offs between melanin-based thermoregulation and other physiological processes, such as metabolic rate or reproductive fitness? These questions highlight the need for longitudinal studies to examine how melanin contributes to climate-driven evolutionary adaptations in insects^[104–110]^.

The regulation of melanin biosynthesis is a complex process influenced by genetic, hormonal, and environmental factors. Studies have identified JH and ecdysteroids as key regulators of melanin production, particularly during cuticle formation and pigmentation. Additionally, transcription factors such as Egr-1 have been implicated in activating melanogenesis-related genes. Despite these insights, significant gaps in knowledge remain: What are the molecular interactions between hormonal signaling and melanogenesis pathways? How do environmental stressors (e.g., temperature, UV radiation) influence gene expression related to melanin synthesis? Can epigenetic modifications regulate melanin production in response to long-term environmental changes? Future studies integrating genomic and transcriptomic analyses will be crucial for unraveling the precise molecular mechanisms controlling melanin biosynthesis^[111–114]^. Despite significant progress in elucidating melanin biosynthesis, several contradictory findings and mechanistic gaps persist across insect taxa. For instance, while studies in *Drosophila* and *Anopheles* have established clear roles for PO and laccase pathways, similar mechanisms are not consistently observed in Coleopteran and Lepidopteran species, where alternative or redundant enzymatic routes appear to operate. Moreover, the transcriptional regulation of melanin synthesis remains poorly defined. Few studies have identified transcription factors such as Egr-1 or the involvement of JH-responsive elements, yet their downstream targets and functional interactions remain largely speculative. Epigenetic regulation, including histone modification and noncoding RNA–mediated control of melanogenic genes, has been proposed but remains empirically understudied in insects. These knowledge gaps limit our understanding of how environmental cues are integrated into gene regulatory networks that govern melanin production. To address these limitations, future research should incorporate cutting-edge molecular and genomic approaches. Single-cell RNA sequencing could elucidate cell-type–specific expression patterns during the melanization response, while CRISPR/Cas-based gene editing and high-throughput screening could systematically identify key regulators of PO and laccase pathways. Integrating these tools with chromatin accessibility and epigenetic profiling (e.g., ATAC-seq, ChIP-seq) would provide a comprehensive view of how melanin synthesis is orchestrated at multiple regulatory levels. Such approaches are essential to reconcile current inconsistencies and to advance from descriptive to mechanistic models of insect melanogenesis^[27,46,115–117]^.

Melanin has been widely retained across insect taxa due to its multifunctional advantages in immunity, thermoregulation, and environmental adaptation. However, melanin also plays a role in sexual selection. Studies on *Danaus plexippus* (monarch butterflies) suggest that darker pigmentation is linked to higher mating success, potentially serving as a signal of genetic fitness. The evolutionary trade-offs of melanin production remain an important area of study. Increased melanin levels provide immunity and environmental protection, but they may also impose metabolic costs. Future research should explore, how does natural selection balance melanin-related benefits and costs in different ecological niches? What are the evolutionary consequences of reduced melanization in certain insect populations? Can melanin production be artificially manipulated to enhance insect resistance against pathogens or environmental stressors? These questions are particularly relevant for vector control strategies, where understanding melanin-based immunity could lead to new approaches for managing insect-borne diseases^[1,87,118–121]^. Beyond its fundamental biological roles, melanin research holds promising implications for applied entomology and pest management. Insights into melanin-mediated immunity could guide the development of novel biological control agents or melanin-targeted inhibitors to reduce pest resistance. For instance, manipulating PO activity or melanization pathways may enhance the susceptibility of agricultural pests to microbial or fungal biocontrols. Additionally, understanding melanin’s role in environmental stress tolerance could inform strategies to predict insect resilience under climate change, aiding in conservation planning and biodiversity protection. In beneficial insects such as pollinators, promoting optimal melanin balance might improve stress resistance and longevity, contributing to ecosystem stability and sustainable agriculture^[21,122–124]^.

Emerging computational tools in structural biology, particularly AI-driven models such as AlphaFold, offer transformative potential for advancing insect melanin research. Despite extensive biochemical characterization, the three-dimensional structures of key melanogenic enzymes – PO, tyrosinase, and laccase – remain incompletely resolved in most insect species, limiting detailed understanding of their catalytic mechanisms, substrate interactions, and regulation. AI-based protein prediction systems, notably AlphaFold (PMID: 39369244), have achieved near-experimental accuracy in modeling protein tertiary structures, enabling unprecedented insights into active site geometry, substrate binding, and potential regulatory motifs. Integrating AlphaFold predictions with experimental enzymology could reveal structure–function relationships central to melanization, guiding the design of selective inhibitors or enhancers that target melanin biosynthetic pathways. Such structure-based approaches may facilitate the development of eco-friendly insecticides or immune-modulatory agents, offering novel tools for pest management and vector control. Moreover, combining AI-based modeling with molecular ecology and omics data would promote a holistic understanding of melanin’s adaptive versatility across taxa. This convergence of computational biology and insect physiology represents a promising paradigm for future research, expanding the scope of melanin studies from biochemical mechanisms to predictive, structure-informed applications in ecological and applied entomology^[125–130]^.

## Future directions

Despite decades of research, many aspects of insect melanization remain incompletely understood. Future investigations should prioritize the integration of multi-omics approaches genomics, transcriptomics, proteomics, and metabolomics to unravel the regulatory networks controlling melanin biosynthesis across taxa. Comparative genomic studies among Diptera, Lepidoptera, and Coleoptera could elucidate lineage-specific adaptations in enzymatic pathways and reveal conserved regulatory motifs. Mechanistic studies are particularly needed to dissect the transcriptional and epigenetic regulation of melanin synthesis. Single-cell RNA sequencing and ATAC-seq analyses can identify cell-type–specific regulatory elements, while CRISPR/Cas9 screening will help functionally validate candidate genes involved in PO and laccase activation. These approaches will clarify how insects balance immune efficiency with oxidative cost and developmental timing. Computational modeling and AI-driven protein structure prediction, such as AlphaFold, present transformative opportunities to elucidate enzyme structures, predict active-site conformations, and design selective inhibitors or activators of melanin biosynthesis. Such applications could inform eco-friendly pest control strategies or enhance disease resistance in beneficial insects. Finally, integrating molecular data with ecological and evolutionary frameworks will be essential to understand how melanin contributes to adaptation under climate change. Longitudinal field studies combined with controlled laboratory experiments could reveal how temperature, UV radiation, and humidity influence the evolution of melanin-related traits. These future directions collectively offer a roadmap for advancing melanin research from descriptive understanding to predictive, mechanistically grounded, and application-oriented science^[131–134]^.

## Conclusion and future directions

This review highlights the multifaceted roles of melanin in insect biology, with functions extending beyond pigmentation to immune defense, thermoregulation, and environmental adaptation. Key findings include the following: PO activation via clip-domain serine proteases is essential for pathogen encapsulation and oxidative stress defense; melanin-mediated thermoregulation enhances survival in cold and arid environments, reducing desiccation and improving heat absorption; hormonal and genetic regulation of melanin production remains an active research area, with JHs and transcription factors playing key roles; and evolutionary trade-offs between melanin production and metabolic costs suggest complex adaptive strategies across insect taxa. Despite these advancements, several critical knowledge gaps remain, Mechanistic pathways linking melanin biosynthesis to environmental stressors remain poorly understood. Epigenetic and genetic regulation of melanogenesis across different insect species needs further investigation. Long-term ecological consequences of melanin variation require comprehensive field studies. Potential applications of melanin research in pest management and disease control remain underexplored. Future research should integrate multi-omics approaches (genomics, transcriptomics, proteomics) with ecological and evolutionary studies to provide a more holistic understanding of melanin’s role in insect adaptation and survival. By addressing these gaps, we can further elucidate the biological significance of melanin and its potential applications in vector control, ecological conservation, and evolutionary biology^[15,135–138]^. From an applied perspective, elucidating melanin biosynthesis and regulation may inform new strategies for pest suppression and environmental conservation. Future research integrating molecular entomology with agricultural biotechnology could enable targeted interventions such as gene-silencing approaches to disrupt melanin-mediated pathogen resistance in pests, or selective breeding to enhance thermotolerance in beneficial insects.

## Data Availability

All data generated or analyzed during this study are included in this published article.
